# Mechanical Overloading Induced-Activation of mTOR Signaling in Tendon Stem/Progenitor Cells Contributes to Tendinopathy Development

**DOI:** 10.3389/fcell.2021.687856

**Published:** 2021-07-12

**Authors:** Daibang Nie, Yiqin Zhou, Wang Wang, Jianying Zhang, James H.-C. Wang

**Affiliations:** ^1^Department of Immunology, College of Basic Medicine, Chongqing Medical University, Chongqing, China; ^2^MechanoBiology Laboratory, Department of Orthopaedic Surgery, University of Pittsburgh, Pittsburgh, PA, United States; ^3^Department of Orthopaedics, Shanghai Changzheng Hospital, Naval Medical University, Shanghai, China; ^4^Department of Physical Medicine and Rehabilitation, University of Pittsburgh, Pittsburgh, PA, United States; ^5^Department of Bioengineering, University of Pittsburgh, Pittsburgh, PA, United States

**Keywords:** mechanical loading, treadmill running, tendon stem cells, mTOR, rapamycin

## Abstract

Despite the importance of mechanical loading in tendon homeostasis and pathophysiology, the molecular responses involved in the mechanotransduction in tendon cells remain unclear. In this study, we found that *in vitro* mechanical loading activated the mammalian target of rapamycin (mTOR) in rat patellar tendon stem/progenitor cells (TSCs) in a stretching magnitude-dependent manner. Application of rapamycin, a specific inhibitor of mTOR, attenuated the phosphorylation of S6 and 4E-BP1 and as such, largely inhibited the mechanical activation of mTOR. Moreover, rapamycin significantly decreased the proliferation and non-tenocyte differentiation of PTSCs as indicated by the reduced expression levels of LPL, PPARγ, SOX-9, collagen II, Runx-2, and osteocalcin genes. In the animal studies, mice subjected to intensive treadmill running (ITR) developed tendon degeneration, as evidenced by the formation of round-shaped cells, accumulation of proteoglycans, and expression of SOX-9 and collagen II proteins. However, daily injections of rapamycin in ITR mice reduced all these tendon degenerative changes. Collectively, these findings suggest that mechanical loading activates the mTOR signaling in TSCs, and rapamycin may be used to prevent tendinopathy development by blocking non-tenocyte differentiation due to mechanical over-activation of mTOR in TSCs.

## Introduction

Tendons are connective tissues that transmit mechanical force (i.e., muscle contraction) to bone to enable joint movement. While a physiological level of mechanical loading is necessary to maintain tendon homeostasis, excessive loading makes tendon tissues susceptible to the development of tendinopathy which is characterized by inflammation and/or degeneration (Maffulli et al., 1998). Tendinopathy is especially prevalent in both occupational and athletic settings that involve repetitive motions (Cook and Purdam, 2009; Zhang and Wang, 2010c) and represents a major healthcare problem in the US.

Like other connective tissues, tendons contain stem/progenitor cells (Bi et al., 2007; Zhang and Wang, 2010a). These tendon stem cells (TSCs) possess common adult stem cell characteristics, and they play a crucial role in tendon development, homeostasis, and repair (Popov et al., 2015; Zhang et al., 2019). We showed that excessive mechanical loading can cause aberrant differentiation of TSCs (Zhang and Wang, 2010b). Others also showed that mechanical loading increases bone morphogenetic protein-2 (BMP-2) expression in TSCs, which in turn promoted osteogenic differentiation of TSCs (Rui et al., 2011). Also, repetitive mechanical loading induces high levels of prostaglandin E2 (PGE_2_) production in tendons, which may induce the differentiation of TSCs into non-tenocytes (Zhang and Wang, 2010c). Years of research have generally concluded that excessive mechanical loading might lead to the development of degenerative tendinopathy commonly seen in clinical settings (Soslowsky et al., 2000; Glazebrook et al., 2008; Abraham et al., 2011; Ng et al., 2011; Zhang and Wang, 2013).

As a central controller of organ growth and development, the mammalian target of rapamycin (mTOR) has been linked to mechanical overloading-induced hypertrophy in the skeletal muscles as well as chondrogenesis (Goodman et al., 2010; Guan et al., 2014; Yoon, 2017). mTOR functions as a master sensor of growth factors, stress, energy status, oxygen, and nutrients, and is involved in a number of major cellular processes. Previous studies showed that rapamycin, a specific inhibitor of mTOR, suppresses the differentiation of human mesenchymal stem cells and primary mouse bone marrow stromal cells to osteoblasts (Singha et al., 2008; Pantovic et al., 2013). A recent study has shown that repetitive mechanical stretching of human tendon cells activates mTOR pathway and increases mRNA translation and collagen synthesis (Mousavizadeh et al., 2020). However, mTOR signaling in TSCs under various mechanical loading conditions remains unclear. In this study, we investigated whether mechanical loading could activate the mTOR signaling in TSCs and explored whether inhibiting mTOR by rapamycin could prevent tendinopathy development due to mechanical overloading placed on the tendon.

## Materials and Methods

### Isolation of Patellar TSCs (PTSCs) and Cell Culture

PTSCs were isolated from the patellar tendons of Sprague-Dawley rats (female, 5 months, Jackson Lab, Bar Harbor, ME) as previously described (Zhang and Wang, 2010a). The protocol for use of the rats was approved by the IACUC of the University of Pittsburgh. Briefly, tendon sheaths from the patellar tendons were removed to obtain the core portions of the tendons. Tendon samples were then minced into small pieces and each 100 mg wet tissue sample was digested in 1 ml phosphate buffered saline (PBS) containing 3 mg collagenase type I (GIBCO, Grand Island, NY, United States) and 4 mg dispase (GIBCO, Grand Island, NY, United States) at 37°C for 1 hr. After centrifugation at 3,500 rpm/min for 15 min to remove the enzymes, the cells were cultured in a growth medium containing DMEM (Lonza, Walkersville, MD, United States) supplemented with 20% fetal bovine serum (FBS; Atlanta Biologicals, Lawrenceville, GA, United States), 100 U/ml penicillin and 100 μg/ml streptomycin (Atlanta Biologicals, Lawrenceville, GA, United States). In all culture experiments, PTSCs at passage 2 or 3 were used.

### Cell Stretching Experiments

We used a uniaxial cell stretching system we had developed previously to investigate the mechanobiological response of rat PTSCs *in vitro* (Wang and Grood, 2000). After PTSCs were seeded in silicone dishes at a density of 4 × 10^5^/dish and cultured in growth medium (DMEM + 10% FBS) overnight, cyclic stretching at 4 and 8% at 0.5 Hz was applied to silicone dishes for 2 h. Control PTSCs without stretching were cultured in the silicone dishes with the same medium. Similar stretching experiments were also done in serum-free medium. A total of three dishes for each loading condition and control were used, respectively, and the experiment was performed in triplicates. After the end of the cell stretching experiments, PTSCs were collected for Western analysis and qRT-PCR.

### Cell Proliferation Assay

CCK-8 assays were performed to assess the PTSC proliferation according to the manufacturer’s instruction (Sigma, St. Louis, MO, United States). Briefly, 1 × 10^3^ cells/well were plated in 96-well plates in 100 μl growth medium and incubated at 37°C for 24 h to allow attachment. The medium was then replaced with fresh DMEM supplemented with 10% FBS, plus the addition of 500 nM rapamycin (Sigma, St. Louis, MO, United States). After 72 h, 10 μl of CCK-8 was added into each well and incubated at 37°C for 2 h. Finally, the absorbance was recorded at 450 nm using a microplate reader (SpectraMax M5, Molecular Devices, CA, United States).

### *In vitro* Differentiation Experiments

The multi-differentiation potential of the PTSCs was tested *in vitro* for adipogenesis, chondrogenesis, and osteogenesis. PTSCs were seeded in a 6-well plate at a density of 2.4 × 10^5^ cells/well and incubated overnight to allow cell attachment. The next day, the media were changed to the respective differentiation cocktails with or without 500 nM rapamycin. Commercially available differentiation cocktails used were StemPro^®^ Adipogenesis, Osteogenesis, and Chondrogenesis Differentiation Kits (Life Technologies, Carlsbad, CA, United States). After 14 days, the adipogenesis was evaluated using Oil Red O staining assay, the chondrogenesis was detected using Alcian blue staining assay, and the osteogenesis was assessed by Alizarin Red S assay (Zhang and Wang, 2010a). All the stained cells were imaged with an inverted microscope (Nikon eclipse, TE2000-U, United States).

### Mouse Treadmill Running Experiments

C57BL/6J mice (female, 3 months old, Jackson Lab, Bar Harbor, ME) were divided into four groups with 6 mice per group. The mice in group 1 were allowed regular cage activities (Cage). The mice in group 2 received a daily IP injection of rapamycin (5 mg/kg body weight) for 12 weeks (Cage + Rapa). The mice in group 3 were subjected to the intensive treadmill running (ITR) at 15 m/min for 3 h/day, 5 days/week for 12 weeks. The mice in group 4 received daily IP injection of rapamycin, with the same dosage as in group 2 (ITR + Rapa), and ran the same ITR. All mice were sacrificed after 12 weeks of experiments. The patellar tendon tissues were harvested from these mice and processed for histochemical and immunohistochemical analyses.

### Histochemical and Immunohistochemical Analyses on Mouse Tendon Tissue Sections

Each mouse patellar tendon was dissected from the knee and collected without skin. The tissue samples were fixed with 4% paraformaldehyde overnight at room temperature, washed three times with PBS, and then soaked in 30% sucrose in PBS at 4°C overnight. The treated tissue samples were embedded in O.C.T compound (Sakura Finetek United States Inc., Torrance, CA, United States) in disposable molds and frozen at −80°C. Then, cryostat sectioning was performed at −25°C to obtain about 8 μm thick tissue section slides, which were left at room temperature overnight. The tissue slides were collected and numbered continuously from the surface to the inside. Consecutive tissue slides were used for H&E, Safranin O and Fast Green, and Masson trichrome staining. Thus, three tissue slides that were numbered 10, 20, 30 in each patellar tendon were stained with H&E, and another three tissue sections numbered 11, 21, 31 in each patellar tendon were stained with Safranin O and Fast Green. In addition, three tissue sections numbered 12, 22, 32 in each patellar tendon were stained with Masson trichrome kit (Sigma-Aldrich, Cat# HT15) according to the standard protocols.

For immunostaining, the section slides with the same number ID from each group were then incubated with rabbit anti-collagen II antibody (1:500, Abcam Cat# ab34712) overnight at 4°C. For SOX-9 staining, the tissue sections were further treated with 0.1% Triton X-100 for 30 min at room temperature, washed with PBS three times, and then the sections were incubated overnight at 4°C with rabbit anti-SOX-9 antibody (1:500, Millipore, Cat# AB5535). The next morning, the tissue sections were washed 3 times with PBS and incubated at room temperature for 2 h with Cy3-conjugated goat anti-rabbit IgG antibody (1:500, Millipore, Cat# AP132C). Finally, the total cell numbers in the tendon tissue sections were analyzed by staining with 4.6-diamidino-2-phenylindole (DAPI), and the stained results were determined under a fluorescent microscope (Nikon, Eclipse TE2000U, United States).

### Quantitative Real-Time RT-PCR (qRT-PCR)

Total RNA was extracted from PTSCs using RNeasy Mini Kit (Qiagen, Valencia, CA, United States). First-strand cDNA was synthesized in a 20 μl reaction from 1 μg total RNA by reverse transcription with SuperScript II (Invitrogen, Carlsbad, CA, United States). The conditions for the cDNA synthesis were 65°C for 5 min and cooling 1 min at 4°C, then 42°C for 50 min and 72°C for 15 min. The qRT-PCR was carried out using QIAGEN QuantiTect SYBR Green PCR Kit (Qiagen, Valencia, CA, United States). Rat-specific primers used for RT-PCR were: PPARγ: 5′-GCCTGCGTCCCCGCCTTAT-3′ (forward), 5′-GCCTGCGTCCCCGCCTTAT-3′ (reverse); LPL: 5′-CTTAAGTGGAAGAACGACTCCTACT-3′ (forward), 5′-GTCATGGCATTTCACAAACACTGCCA-3′ (reverse) (Mello et al., 2008); SOX-9: 5′- AGCGACAACTTTACCAG-3′ (forward), 5′-GGAAAACAGAGAACGAAAC-3′ (reverse); Collagen II: 5′-GGCTTAGGGCAGAGAGAGAAG-3′ (forward), 5′-TGGACAGTAGACGGAGGAAAGTC-3′ (reverse) (Rafiei and Ashrafizadeh, 2018); Runx-2: 5′-CCGCACGACAACCGCACCAT-3′ (forward), 5′-CGCTCCGCTTC-3′ (reverse) (Yoshida et al., 2004); and Osteocalcin: 5′-AAAGCCCAGCGACTCT-3′ (forward), 5′-CTAAACGGTGGTGCCATAGAT-3′ (reverse) (Rafiei and Ashrafizadeh, 2018). GAPDH was used as an internal control. All primers were synthesized by Invitrogen (Carlsbad, CA, United States). After an initial denaturation for 10 min at 95°C, PCR was performed for 30 cycles for GAPDH, and 40 cycles for LPL, PPARγ, SOX-9, collagen II, Runx-2, and osteocalcin, with each cycle consisting of denaturation for 50 s at 95°C, followed by annealing for 30 s at 58°C for all the genes. At least three independent experiments were performed to obtain relative expression levels of each gene. Data were analyzed by the 2^–ΔΔ^ ^*Ct*^ method (Livak and Schmittgen, 2001). The gene expression levels of the treatment groups were normalized to that of the control group for each of the three experiments.

### Western Blot Analysis

Cell lysates were prepared in RIPA buffer using standard procedures provided by the manufacturer (Sigma, St. Louis, MO, United States). The protein concentrations were measured using a BCA Protein Assay Kit (Thermo Fisher Scientific, Pittsburgh, PA, United States) to ensure equal loading. Loading buffer was added to 30 μg protein, and samples were heated at 100 ℃ for 5 min before separated on 4–20% SDS-PAGE gels, then transferred onto PVDF membranes (Bio-Rad, Hercules, CA, United States). Protein blots were blocked with 5% Non-Fat dry milk (Bio-Rad, Hercules, CA, United States) at room temperature for 1 h. Antibodies used were p-S6 (Cell Signaling Technology, Cat# 4858), S6 (1:1,000, Cell Signaling Technology, Cat# 2317), p-4EBP1 (1:1,000, Cell Signaling Technology Cat# 2855), 4EBP1 (1:1,000, Cell Signaling Technology, Cat# 9644), and β-actin (1:10,000, Abcam, Cat# ab8226). The next day, the blots were washed three times with 0.1% Tween 20-containing PBS buffer (PBS-T) buffer, then incubated with the corresponding secondary antibodies (1:15,000, LI-COR Biosciences) for 1 h at room temperature. Following another three washes with PBS-T buffer, visualization of the protein bands of the blots was realized with the LiCoR Odyssey imager (LI-COR Biosciences, Lincoln, NE, United States), and semi-quantification of the protein bands was done using the software provided by the LiCoR Odyssey imager.

### Semi-Quantification of Histochemical and Immunohistochemical Staining Results

Semi-quantification was performed to quantify the extent of cell marker staining in tendon tissue sections. Three tissue sections/mouse from 6 mice/group with a total of 18 tissue sections were stained. The positive staining in the sections was identified under the microscope and analyzed using SPOT imaging software (Diagnostic Instruments, Inc., Sterling Heights, MI). The proportion of positive staining from histochemical staining was determined by dividing the positively stained area by the total area viewed under the microscope. For immunohistochemical staining, the proportion of positive staining was calculated by dividing the positively stained cell numbers by the total cell numbers. The mean value from all 18-tissue section staining results represented the final percentage of positive staining.

### Statistical Analysis

All data were represented by the mean and standard deviation (mean ± SD). One-way analysis of variance (ANOVA) was used for statistical data analysis. For multiple comparisons, the Fisher’s LSD *post hoc* test was performed. All statistical tests were done using GraphPad Prism 7 (GraphPad Software, San Diego, CA). Differences with a *p* < 0.05 were considered statistically significant.

## Results

### Mechanical Loading Activates the mTOR Signaling in PTSCs *in vitro*

**FIGURE 1:**
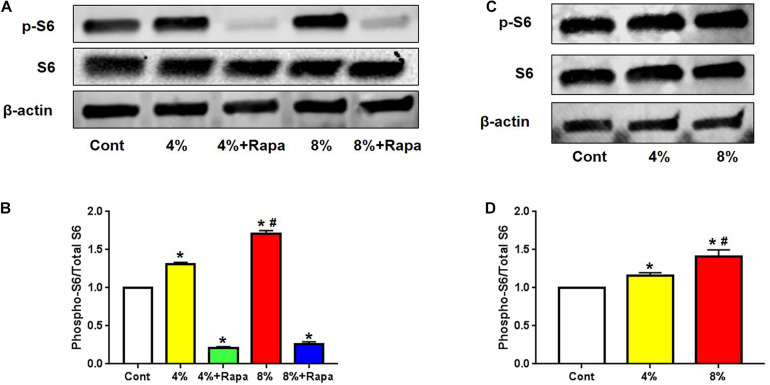
Mechanical loading activates mTOR in a stretching magnitude-dependent manner and rapamycin abolishes the loading-induced activation. Western blot analysis shows higher levels of p-S6 in 4 and 8% stretched PTSCs. The addition of rapamycin to the culture medium largely blocks this effect **(A)**. Quantification of the Western blot results shows that 4 and 8% stretching significantly increase p-S6 levels, but rapamycin negates this effect **(B)**. Western blot analysis of p-S6 levels in PTSCs subjected to 4 and 8% mechanical stretching and cultured in medium without FBS shows that p-S6 levels are higher in stretched cells than the control cells **(C)**. Quantification of the Western blot results confirms these findings **(D)**. Control (Cont): PTSCs cultured under the same culture conditions as other groups but without stretching. Note that * denotes 4 and 8% compared to control, # denotes 8% + Rapa compared to 8% stretch in **(B)**, * denotes 4 and 8% compared to control, # denotes 8% compared to 4% stretch in **(D)** (*n* = 3, and values are mean ± SD. *p < 0.05, ^#^*p* < 0.05).

To investigate whether mechanical loading could activate mTOR signaling in PTSCs, we isolated TSCs from the rat patellar tendons. These PTSCs were subjected to different levels of cyclic stretching to mimic normal (4%) and excessive (8%) mechanical loads on tendon tissue. We found that both 4 and 8% stretching increased the expression of phospho-S6, compared to the control PTSCs cultured in the same medium but without stretching (Figure 1A). Such an increase in mTOR signaling activity appeared to be positively correlated to the magnitude of mechanical stretching (Figure 1B). However, the addition of rapamycin resulted in decreased p-S6/S6, and p-4E-BP1/4E-BP1, suggesting rapamycin inhibited loading-induced activation of the mTOR (Figure 1 and Supplementary Figure 1). This indicates that mechanical loading activates mTOR signaling in a loading magnitude-dependent manner, and such mechanical activation of mTOR can be inhibited by rapamycin treatment.

### Mechanical Activation of mTOR Is Independent of FBS *in vitro*

Given the role of mTOR as a master sensor of cellular conditions including stress and metabolic substrates, we determined whether those nutrient factors in FBS could affect mechanical loading-induced mTOR activation. We cultured PTSCs in medium without FBS and subjected them to 4 or 8% stretching. Quantification of Western blot results showed that the increase in the ratio of p-S6/S6 protein levels under 4 and 8% stretching without FBS in medium (Figures 1C,D) was similar to that when the cells were stretched at 4 and 8% with the presence of FBS (Figures 1A,B). The results suggest that mechanical loading-induced mTOR activation is likely independent of those factors (e.g., growth factors and nutrients) in FBS.

### Rapamycin Decreases PTSC Proliferation and Differentiation *in vitro*

**FIGURE 2:**
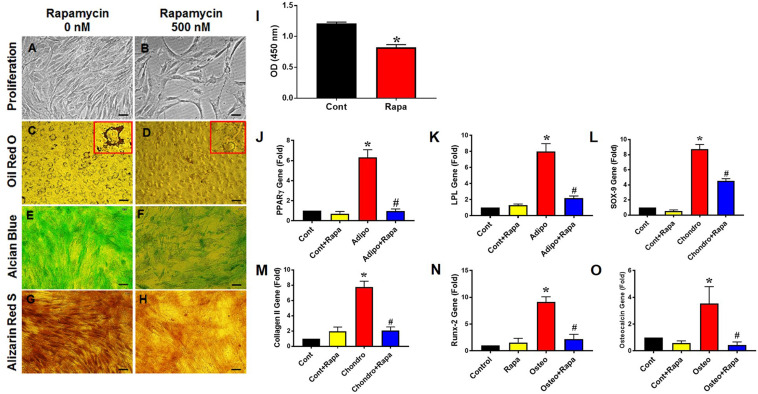
Rapamycin inhibits PTSC proliferation and differentiation *in vitro*. Rapamycin at the dose 500 nM significantly reduces PTSC proliferation (**I**; see also decreased cell density in **B** compared to **A**). Similarly, rapamycin inhibits PTSC differentiation in the adipogenic (**C,D**; see large amounts of lipid droplets in the inset of **C** but not in **D**), chondrogenic **(E,F**), and osteogenic **(G,H)** induction media. Moreover, the rapamycin treatment inhibits the expression of marker genes: PPARγ **(J)** and LPL **(K)** for adipocytes, SOX-9 **(L)** and collagen II **(M)** for chondrocytes, and Runx-2 **(N)** and osteocalcin **(O)** for osteocytes. Note that * denotes treatments compared to control, # denotes comparison between each differentiation medium treatment + Rapa with each medium treatment alone (*n* = 3, and values are mean ± SD. *p < 0.05, ^#^*p* < 0.05). Black bars: 50 μm.

To determine the effect of rapamycin on PTSC proliferation and differentiation, we utilized rat PTSCs under normal culture conditions. We found that the treatment of rapamycin for 72 h significantly reduced PTSC proliferation compared to the control (Figures 2A,B,I). PTSCs treated with 500 nM of rapamycin maintained good viability in the rapamycin treated group, with slight morphological changes compared to the control group (Figures 2A,B). Next, we cultured rat PTSCs in three separate differentiation media to test their potentials to undergo adipogenesis, chondrogenesis, and osteogenesis. PTSCs in the control group without rapamycin were able to differentiate into adipocytes, chondrocytes, and osteocytes, as shown by the staining of Oil Red O, Alcian blue, and Alizarin Red S assay, respectively (Figures 2C,E,G). However, the presence of rapamycin (500 nM) in the osteogenic, adipogenic, or chondrogenic induction media markedly reduced the extent of TSC differentiation (Figures 2D,F,H). These results were also supported by gene expression results of those differentiation markers: PPARγ and LPL for adipogenesis, SOX-9 and collagen II for chondrogenesis, and Runx-2 and osteocalcin for osteogenesis (Figures 2J–O).

### Rapamycin Blocks Mechanical Overloading-Induced Non-tenocyte Differentiation of PTSCs

**FIGURE 3:**
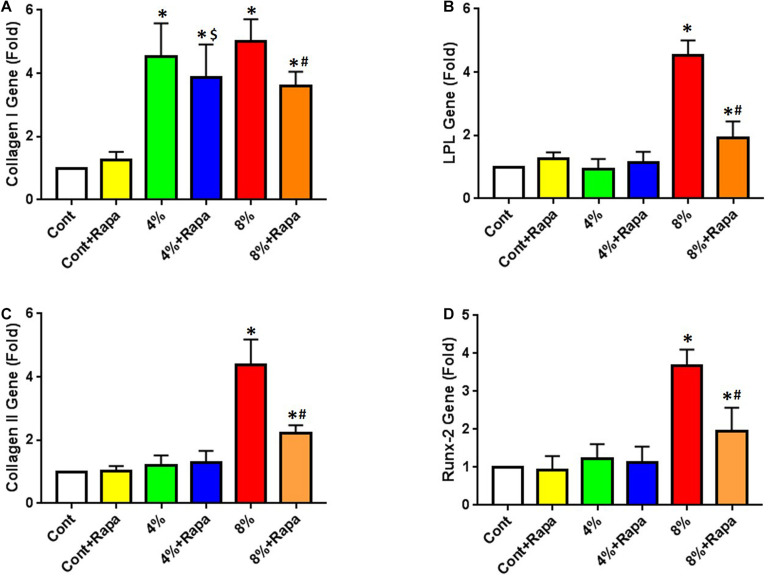
Rapamycin inhibits non-tenocyte differentiation of PTSCs induced by mechanical stretching. Rapamycin does not significantly decrease the collagen I expression induced by 4% stretching (4% + Rapa vs. 4%), but it does decrease the collagen I expression at 8% stretching (8% + Rapa vs. 8%) **(A)**. Moreover, rapamycin treatment significantly reduces the expression of non-tenocyte related genes induced by 8% stretching, including LPL for adipocytes **(B)**, collagen II for chondrocytes **(C)**, and Runx-2 for osteocytes **(D)**. Under 4% stretching, however, rapamycin does not cause changes in the expression of these non-tenocyte genes **(B–D)**. Note that * denotes 4 and 8% compared to control, $ denotes 4% + Rapa compared to 4%, and # denotes 8% + Rapa compared to 8% (*n* = 3, and values are mean ± SD. *p < 0.05, ^#^*p* < 0.05).

Next, we tested whether rapamycin could rescue mechanical loading induced non-tenocyte differentiation of PTSCs in an *in vitro* cell stretching model. We cultured cells in a normal culture medium (DMEM plus 10% FBS) and subjected cells to 4 or 8% stretching. We found that after stretching, the expression of the tenocyte-related gene collagen I was up-regulated in PTSCs (Figure 3A). Although rapamycin appeared to decrease collagen I expression induced by 4% stretching, there was no significant difference between the 4% stretching group and the 4% stretching plus rapamycin treated group (Figure 3A). There was also no significant difference in non-tenocyte-related gene expression in PTSCs between these two groups. However, rapamycin significantly decreased 8% stretching-induced collagen I levels in PTSCs (Figure 3A). Furthermore, 8% stretching of PTSCs significantly increased the expression of non-tenocyte-related genes, including LPL for adipocytes (Figure 3B), collagen II for chondrocytes (Figure 3C), and Runx-2 for osteocytes (Figure 3D), as compared to the control group. Finally, the addition of rapamycin significantly reduced the expression of all three non-tenocyte marker genes.

### Rapamycin Inhibits ITR-Induced Tendon Degeneration *in vivo*

**FIGURE 4:**
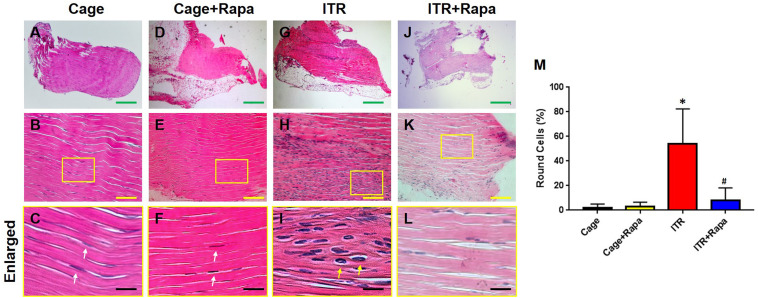
Rapamycin blocks the ITR-induced cellular morphological changes in mouse patellar tendons. H&E staining shows the normal elongated shape of the tendon cells in cage control **(A–C)** and rapamycin injected mouse tendons (**D–F**, white arrows in **C,F**). Many round shape cells are present in ITR tendons (**G-I**; yellow arrows in **I**). With rapamycin injection prior to ITR, markedly fewer round shaped cells are shown in the tendon tissues **(J–L)**. Semi-quantification analysis indicates that more than 55% of the cells in ITR tendon, but less than 10% of the cells in ITR + Rapa treated tendons are round **(M)**. Note that * denotes ITR compared to control, # denotes ITR + Rapa compared to ITR (*p < 0.05, ^#^*p* < 0.05). Green bar: 500 μm; Yellow bars: 100 μm; Black bars: 25 μm.

Furthermore, we tested whether rapamycin could rescue mechanical loading induced tendon degeneration in an ITR mouse model. Histochemical analysis showed that the mouse tendon cells in the cage group exhibited an elongated morphology (Figures 4A–C, white arrows in Figure 4C) and rapamycin injection group (Figures 4D–F, white arrows in Figure 4F). However, the majority of the patellar tendon cells changed into a round shape after mice were subjected to an ITR regimen for 12 weeks (Figures 4G–I, yellow arrows in Figure 4I). This morphological alteration was, however, blocked by rapamycin injections (Figures 4J–L). Semi-quantification of the results indicated that more than 55% of the cells in ITR tendons were round-shaped cells, but only less than 10% cells were round-shaped in the rapamycin treated ITR tendons (Figure 4M).

**FIGURE 5:**
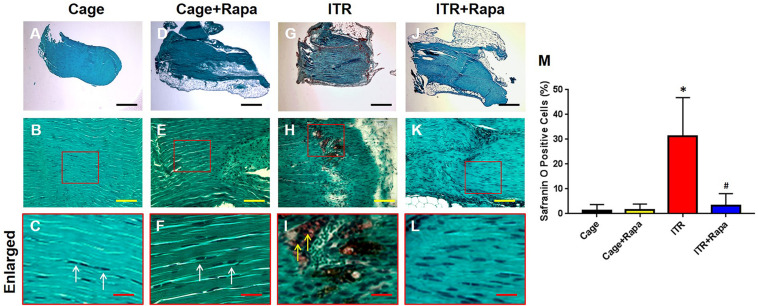
Rapamycin inhibits ITR-induced degenerative changes in mouse tendons. Histochemical analysis by Safranin O and Fast Green staining shows tendon cells in elongated shape in collagen tissues from tendons of the cage control (**A–C**, white arrows in **C**) and tendons with rapamycin injections (**D–F**, white arrows in **F**). However, many round-shaped cells are positively stained with Safranin O in tendons of the ITR group (red in **G,H,I**; yellow arrows in **I**). Rapamycin injection blocks the ITR-induced degenerative changes in the tendons **(J–L)**. Semi-quantification analysis indicates that more than 30% of the cells in ITR tendon are positively stained with Safranin O, but less than 7% of the cells in ITR + Rapa treated tendons are positively stained with Safranin O **(M)**. Note that * denotes ITR compared to control, # denotes ITR + Rapa compared to ITR (*p < 0.05, ^#^*p* < 0.05). Black bars: 500 μm; Yellow bars: 100 μm; Red bars: 25 μm.

Similar findings were obtained with the Safranin O and Fast Green staining. Tendon cells in cage control mice (Figures 5A–C) and rapamycin injection mice (Figures 5D–F) displayed a normal appearance with an elongated shape (Figures 5A–F, white arrows in Figures 5C,F). However, tendons under the ITR condition were positively stained with Safranin O, indicating that non-tenocyte differentiation of PTSCs likely took place (red in Figures 5G–I, yellow arrows in Figure 5I) and as a result, proteoglycans were accumulated in the tendon matrix. Such tendon degeneration due to ITR was effectively prevented by rapamycin injections, with the corresponding tendon tissues showing a lack of Safranin O staining (Figures 5J–L). Semi-quantification of the staining results showed that more than 32% of the tendon cells in ITR tendons were positively stained with Safranin O, but less than 5% of the tendon cells in rapamycin treated ITR tendons were positively stained (Figure 5M).

**FIGURE 6:**
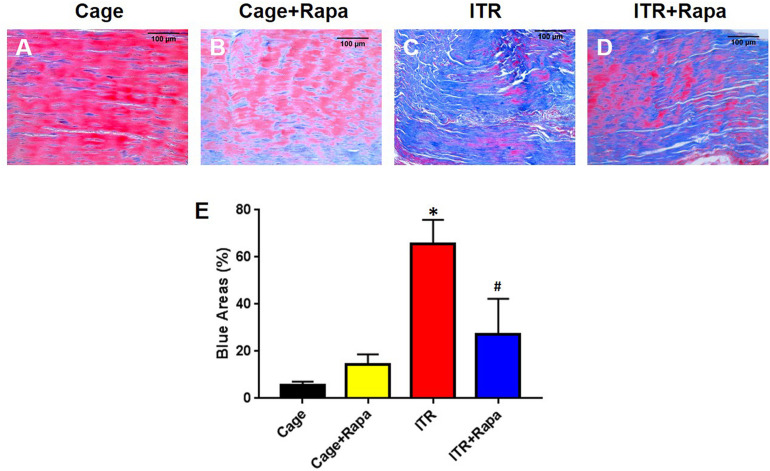
Rapamycin reduces ITR-induced tendon tissue degeneration. Masson trichrome staining results indicate that the collagen fascicles in the patellar tendons of cage control mice are dense connective tissues, which are well organized, and positively stained with Biebrich scarlet-acid fuchsin (red in **A**). Similar results are found in the tendon tissues of the mice treated with rapamycin injection daily for 12 weeks **(B)**. However, in ITR mouse tendons, loose and disorganized connective tissues are present and positively stained with aniline blue **(C)**. Finally, rapamycin injection reduces the degenerative changes in mouse tendons induced by ITR, as shown by decreased blue area and increased the red area **(D)**. Semi-quantification analysis confirms these findings **(E)**. Note that * denotes ITR compared to control, # denotes ITR + Rapa compared to ITR (*p < 0.05, ^#^*p* < 0.05). Black bars: 100 μm.

Additionally, Masson trichrome (MT) staining results showed that the mechanical overloading by ITR caused degenerative changes in tendon tissue, as evidenced by the loose, disorganized connective tissue that was strongly stained blue (Figure 6C). However, the cage control tendons (Figure 6A) and rapamycin injection only tendons (Figure 6B), which consist of normally dense connective tissues (mainly tight collagen fiber bundles), were largely stained red (Figures 6A,B,E). Rapamycin injection reduced degenerative changes in ITR tendons as shown by the decrease in the loose, degenerative tendinous tissues and the increase in the dense, normal-like tendinous tissues (Figure 6D). These results were also supported by semi-quantification analysis (Figure 6E).

**FIGURE 7:**
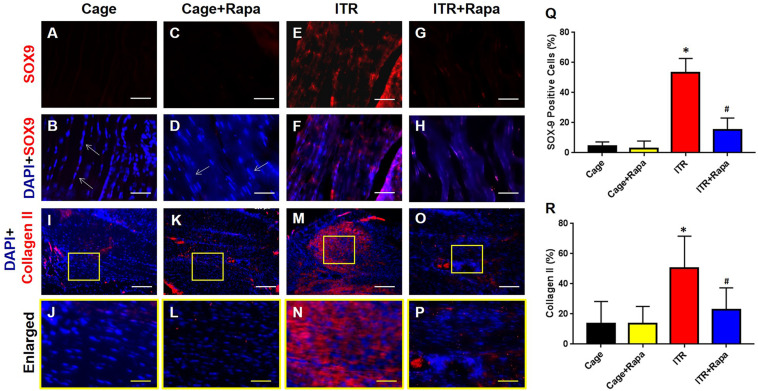
Rapamycin blocks ITR-induced SOX-9 and collagen II expression in mouse tendons. The cells of cage control tendons **(A,B,I,J)** and tendons from the rapamycin injection group **(C,D,K,L)** exhibit an elongated shape with negative SOX-9 staining (white arrows in **B,D**) and minimal levels of collagen II staining **(K,L)**. However, many cells are positively stained with SOX-9 (red cells in **E,F**) and collagen II (red cells in **M,N**) in tendons of the ITR group. Rapamycin injection blocks the ITR-induced degenerative changes in the tendon, as evidenced by markedly fewer cells positively stained with SOX-9 **(G,H)** or collagen II **(O,P)**. Semi-quantification analysis indicates that more than 55% of the cells in ITR tendons are positively stained either with SOX-9 **(Q)** or with collagen II **(R)**. Note that * denotes ITR compared to cage control, # denotes ITR + Rapa compared to ITR (*p < 0.05, ^#^*p* < 0.05). White bars: 200 μm; Yellow bars: 25 μm.

Furthermore, immunostaining confirmed tendinopathy-like changes with high percentages of the tendon cells from the ITR group positively stained with SOX-9 (Figures 7A–H,Q) and collagen II (Figures 7I–P,R). These non-tenocyte markers were undetected within tendon tissues collected from cage mice (Figures 7A,B,I,J) and rapamycin injection only mice (Figures 7C,D,K,L). Thus, rapamycin effectively inhibited the ITR-induced upregulation of SOX-9 expression in the mouse tendons, as evidenced by limited numbers of cells positively stained with these markers in the ITR + Rapa group (Figures 7G,H,Q). Similarly, enhanced collagen type II expression in the tendon tissues by ITR was reverted by rapamycin treatment (Figures 7O,P,R).

## Discussion

Mechanical loading is well recognized for its importance in tendon development, tendon injury healing, and pathology, but tendon mechanotransduction is still poorly understood and represents a key area in tendon research. In this report, we show that mechanical loading activated mTOR signaling in PTSCs, and that treatment with rapamycin suppressed cell proliferation and differentiation *in vitro* and ITR-induced tendon degeneration *in vivo*. Based on the findings from the *in vitro* study, we suggest that mTOR signaling in PTSCs plays a critical role in the development of degenerative tendinopathy due to mechanical overloading in ITR mice, and that as a specific inhibitor of mTOR, rapamycin may be used to prevent tendinopathy development.

Previous studies showed that mechanical stimulation can lead to the activation of the mTOR pathway in human periodontal ligament fibroblasts and human tendon cells (Blawat et al., 2020; Mousavizadeh et al., 2020). Research has shown that mTOR signaling may be necessary for an increase in protein synthesis and resulting hypertrophic tissue in response to mechanical loads (Goodman, 2019). Also, the activation of mTOR signaling is sufficient to induce an increase in muscle protein synthesis and muscle fiber hypertrophy (Goodman et al., 2010). In addition, mechanical activation of mTOR is required for cell proliferation, chondrogenesis, and cartilage growth during bone development (Guan et al., 2014). This study focused on assessing the effect of mTOR activation on PTSCs during normal and excessive mechanical loading, with results suggesting that mTOR activation of PTSCs may be dependent upon the mechanical load (4 vs. 8%), and that mechanical loading itself may result in cellular stress that mTOR is able to sense in tendon tissue.

Excessive mechanical loading is known to cause catabolic changes in tendons and induce differentiation of TSCs into non-tenocytes, which may lead to the development of degenerative tendinopathy frequently seen in clinical settings (Zhang et al., 2010; Zhang and Wang, 2010b; Abraham et al., 2011; Zhang and Wang, 2013). In this study, mechanical over-stretching of PTSCs *in vitro* at 8% caused an increase in non-tenocyte differentiation, including adipogenesis, chondrogenesis, and osteogenesis. However, rapamycin reduced the expression of these non-tenocyte genes. Based on these results, we assessed the preventative potential of the classic mTOR antagonist rapamycin in the prevention of tendinopathy development due to excessive mechanical loading. Indeed, daily injections of rapamycin in mice block the formation of degenerative changes in ITR tendons.

Elevated expression of SOX-9 and collagen II in tendons were observed in mice subjected to ITR, which matched similar results observed in clinical specimens of excessive mechanical loading-induced tendinopathy (Rui et al., 2012). In our data, the presence of these markers was greatly reduced in the tendon tissues treated with rapamycin prior to ITR, suggesting that mTOR signaling due to mechanical loading conditions on tendon cells regulates the expression of these non-tendinous tissue markers, and hence mechanical activation of mTOR may play a crucial role in the development of tendinopathy.

An important observation in this study was with Masson trichrome (MT) staining. Normal cage tendons were stained red, whereas ITR tendons were stained blue. The results are consistent with previous reports that loose, immature, or degenerative connective tissues are stained blue, whereas normal tendon consisting of dense connective tissues (mainly collagen type I) are stained red with MT staining (Martinello et al., 2015; Bastiani et al., 2018). The reason for such a difference in color may be that dye penetration is easier in loose connective tissue than in the normal dense connective tissue (Bastiani et al., 2018). Using MT staining, this study was able to confirm that rapamycin injection decreased the extent of connective tissue disorganization and degeneration in the ITR tendons.

There are a few limitations in this study. First, the detailed upstream and downstream signaling components involved in the mTOR response to mechanical loading were not determined. mTOR signaling has been correlated with the formation of muscle ossification, which was shown to be negatively regulated by PI3Kα (Valer et al., 2019). Therefore, while this study focused on the effects of rapamycin/mTOR signaling on PTSCs *in vitro* and tendons *in vivo* due to mechanical overloading, future studies are warranted to investigate detailed mTOR signaling pathways under mechanical loading conditions. Second, mTOR is known to be linked to aging-associated tendon disorders (Wilkinson et al., 2012; Zaseck et al., 2016), and future studies may include mice of different ages in the ITR model to further elucidate the role of mTOR and the effects of rapamycin in the development of tendinopathy. Third, it is known that mTOR exists in two complexes, namely mTORC1 and mTORC2. mTORC1 regulates protein translation, autophagy, among other functions, and is mediated by S6K1, 4E-BP1, and others. On other hand, mTORC2 is considered mainly as a downstream effector of IGF-1 signaling pathway (Kennedy and Lamming, 2016). Since this study showed increased phosphorylation of S6 and 4E-BP1, it is likely that mechanical loading activated mTORC1 pathway. However, the existing data from this study are not sufficient yet to show that mTORC1 actually mediates mechanical overloading-induced non-tenocyte differentiation of PTSCs. Future research is warranted to determine respective roles of mTORC1 and mTORC2 in the non-tenocyte differentiation of PTSCs and development of tendinopathy due to mechanical overloading conditions.

Fourth, this study mainly utilized histochemical and immunohistochemical analyses to determine the results of ITR-induced tendon degeneration and the protective effects of rapamycin injections. However, other methodologies such as TEM and mechanical testing of tendon are highly desirable to show changes in structural and mechanical properties of the tendon due to mechanical loading and rapamycin treatment. Finally, while the identity of cell type, or PTSCs, in our *in vitro* study was clearly defined, it is not certain what exact types of cells were involved in the differentiation of these cells into chondrocyte-like cells, judged by the round cell shape and expression of those chondrogenic markers (proteoglycan accumulation, and expression of SOX-9 and collagen II proteins). However, PTSCs should be part of the tendon cell population because, unlike terminally differentiated “tenocytes,” these stem/progenitor cells possess multi-differentiation potential (Zhang and Wang, 2010a). Other stem/progenitor cells from paratenon (Zhang et al., 2021) could also be part of the cells that undergo non-tenocyte differentiation in response to mechanical overloading-induced tendon injury.

In conclusion, this study showed that mechanical loading activates mTOR signaling in PTSCs, and rapamycin treatment reduces non-tenocyte differentiation *in vitro.* Moreover, injections of rapamycin decreased the tendon’s degenerative changes in mice subjected to ITR. These findings suggest that rapamycin may be used as a therapeutic option to prevent the development of tendinopathy due to mechanical overloading placed on the tendon.

## Data Availability Statement

The original contributions presented in the study are included in the article/Supplementary Material, further inquiries can be directed to the corresponding author/s.

## Ethics Statement

The animal study was reviewed and approved by the Ethics Committee of the University of Pittsburgh.

## Author Contributions

DN, YZ, and JZ contributed to the cell culture experiments, animal experiments, data acquisition, and analysis. DN and WW drafted the manuscript. JZ took part in the experimental design and manuscript revision. JW conceived the study, supervised experiments, and data analysis, and revised the manuscript. All authors read and approved the final manuscript.

## Conflict of Interest

The authors declare that the research was conducted in the absence of any commercial or financial relationships that could be construed as a potential conflict of interest.

## Supplementary Material

Supplementary Figure 1 Rapamycin inhibits the phosphorylation of both S6 and 4E-BP1.

## References

[B1] Abraham T. Fong G. Scott A. (2011). Second harmonic generation analysis of early Achilles tendinosis in response to in vivo mechanical loading. *BMC Musculoskelet. Disord.* 12:26. 10.1186/1471-2474-12-26 21269488 PMC3045393

[B2] Bastiani G. Corte F. D. Brass K. E. Cantarelli C. Dau S. Kommers G. D. (2018). Histochemistry of equine damaged tendons, ligaments and articular cartilage. *Acta Sci. Vet.* 146:1612.

[B3] Bi Y. Ehirchiou D. Kilts T. M. Inkson C. A. Embree M. C. Sonoyama W. (2007). Identification of tendon stem/progenitor cells and the role of the extracellular matrix in their niche. *Nat. Med.* 13 1219–1227. 10.1038/nm1630 17828274

[B4] Blawat K. Mayr A. Hardt M. Kirschneck C. Nokhbehsaim M. Behl C. (2020). Regulation of Autophagic Signaling by Mechanical Loading and Inflammation in Human PDL Fibroblasts. *Int. J. Mol. Sci.* 21:9446. 10.3390/ijms21249446 33322510 PMC7763506

[B5] Cook J. L. Purdam C. R. (2009). Is tendon pathology a continuum? . *Br. J. Sports Med.* 43 409–416. 10.1136/bjsm.2008.051193 18812414

[B6] Glazebrook M. A. Wright J. R. Jr. Langman M. Stanish W. D. Lee J. M. (2008). Histological analysis of achilles tendons in an overuse rat model. *J. Orthop. Res.* 26 840–846. 10.1002/jor.20546 18183626

[B7] Goodman C. A. (2019). Role of mTORC1 in mechanically induced increases in translation and skeletal muscle mass. *J. Appl. Physiol.* 127 581–590. 10.1152/japplphysiol.01011.2018 30676865

[B8] Goodman C. A. Miu M. H. Frey J. W. Mabrey D. M. Lincoln H. C. Ge Y. (2010). A phosphatidylinositol 3-kinase/protein kinase B-independent activation of mammalian target of rapamycin signaling is sufficient to induce skeletal muscle hypertrophy. *Mol. Biol. Cell* 21 3258–3268. 10.1091/mbc.E10-05-0454 20668162 PMC2938390

[B9] Guan Y. Yang X. Yang W. Charbonneau C. Chen Q. (2014). Mechanical activation of mammalian target of rapamycin pathway is required for cartilage development. *FASEB J.* 28 4470–4481. 10.1096/fj.14-252783 25002119 PMC4202102

[B10] Kennedy B. K. Lamming D. W. (2016). The mechanistic target of rapamycin: the grand conducTOR of metabolism and aging. *Cell Metab.* 23 990–1003. 10.1016/j.cmet.2016.05.009 27304501 PMC4910876

[B11] Livak K. J. Schmittgen T. D. (2001). Analysis of relative gene expression data using real-time quantitative PCR and the 2(-Delta Delta C(T)) Method. *Methods* 25 402–408. 10.1006/meth.2001.1262 11846609

[B12] Maffulli N. Khan K. M. Puddu G. (1998). Overuse tendon conditions: time to change a confusing terminology. *Arthroscopy* 14 840–843. 10.1016/s0749-8063(98)70021-0 9848596

[B13] Martinello T. Pascoli F. Caporale G. Perazzi A. Iacopetti I. Patruno M. (2015). Might the Masson trichrome stain be considered a useful method for categorizing experimental tendon lesions? *Histol. Histopathol.* 30 963–969. 10.14670/HH-11-601 25733060

[B14] Mello T. Nakatsuka A. Fears S. Davis W. Tsukamoto H. Bosron W. F. (2008). Expression of carboxylesterase and lipase genes in rat liver cell-types. *Biochem. Biophys. Res. Commun.* 374 460–464. 10.1016/j.bbrc.2008.07.024 18639528 PMC2566784

[B15] Mousavizadeh R. Hojabrpour P. Eltit F. McDonald P. C. Dedhar S. McCormack R. G. (2020). β1 integrin. *ILK and mTOR regulate collagen synthesis in mechanically loaded tendon cells*. *Sci. Rep.* 10:12644. 10.1038/s41598-020-69267-6 32724089 PMC7387456

[B16] Ng G. Y. Chung P. Y. Wang J. S. Cheung R. T. (2011). Enforced bipedal downhill running induces Achilles tendinosis in rats. *Connect. Tissue Res.* 52 466–471. 10.3109/03008207.2011.562334 21591929

[B17] Pantovic A. Krstic A. Janjetovic K. Kocic J. Harhaji-Trajkovic L. Bugarski D. (2013). Coordinated time-dependent modulation of AMPK/Akt/mTOR signaling and autophagy controls osteogenic differentiation of human mesenchymal stem cells. *Bone* 52 524–531. 10.1016/j.bone.2012.10.024 23111315

[B18] Popov C. Burggraf M. Kreja L. Ignatius A. Schieker M. Docheva D. (2015). Mechanical stimulation of human tendon stem/progenitor cells results in upregulation of matrix proteins, integrins and MMPs, and activation of p38 and ERK1/2 kinases. *BMC Mol. Biol.* 16:6. 10.1186/s12867-015-0036-6 25880261 PMC4373449

[B19] Rafiei H. Ashrafizadeh M. (2018). Expression of collagen Type II and osteocalcin genes in mesenchymal stem cells from rats treated with lead acetate II. *Iranian. J. Toxicol.* 12 35–40. 10.32598/ijt.12.5.540.1

[B20] Rui Y. F. Lui P. P. Ni M. Chan L. S. Lee Y. W. Chan K. M. (2011). Mechanical loading increased BMP-2 expression which promoted osteogenic differentiation of tendon-derived stem cells. *J. Orthop. Res.* 29 390–396. 10.1002/jor.21218 20882582

[B21] Rui Y. F. Lui P. P. Rolf C. G. Wong Y. M. Lee Y. W. Chan K. M. (2012). Expression of chondro-osteogenic BMPs in clinical samples of patellar tendinopathy. *Knee Surg. Sports Traumatol. Arthrosc.* 20 1409–1417. 10.1007/s00167-011-1685-8 21946950

[B22] Singha U. K. Jiang Y. Yu S. Luo M. Lu Y. Zhang J. (2008). Rapamycin inhibits osteoblast proliferation and differentiation in MC3T3-E1 cells and primary mouse bone marrow stromal cells. *J. Cell. Biochem.* 103 434–446. 10.1002/jcb.21411 17516572

[B23] Soslowsky L. J. Thomopoulos S. Tun S. Flanagan C. L. Keefer C. C. Mastaw J. (2000). Neer Award 1999. Overuse activity injures the supraspinatus tendon in an animal model: a histologic and biomechanical study. *J. Shoulder Elbow Surg.* 9 79–84. 10810684

[B24] Valer J. A. Sánchez-de-Diego C. Gámez B. Mishina Y. Rosa J. L. Ventura F. (2019). Inhibition of phosphatidylinositol 3-kinase α (PI3Kα) prevents heterotopic ossification. *EMBO. Mol. Med.* 11:e10567. 10.15252/emmm.201910567 31373426 PMC6728602

[B25] Wang J. H. Grood E. S. (2000). The strain magnitude and contact guidance determine orientation response of fibroblasts to cyclic substrate strains. *Connect. Tissue Res.* 41 29–36. 10.3109/03008200009005639 10826706

[B26] Wilkinson J. E. Burmeister L. Brooks S. V. Chan C. C. Friedline S. Harrison D. E. (2012). Rapamycin slows aging in mice. *Aging Cell* 11 675–682. 10.1111/j.1474-9726.2012.00832.x 22587563 PMC3434687

[B27] Yoon M. S. (2017). mTOR as a key regulator in maintaining skeletal muscle mass. *Front. Physiol.* 8:788. 10.3389/fphys.2017.00788 29089899 PMC5650960

[B28] Yoshida C. A. Yamamoto H. Fujita T. Furuichi T. Ito K. Inoue K. (2004). Runx2 and Runx3 are essential for chondrocyte maturation, and Runx2 regulates limb growth through induction of Indian hedgehog. *Genes Dev.* 18 952–963. 10.1101/gad.1174704 15107406 PMC395853

[B29] Zaseck L. W. Miller R. A. Brooks S. V. (2016). Rapamycin attenuates age-associated changes in tibialis anterior tendon viscoelastic properties. *J. Gerontol. A Biol. Sci. Med. Sci.* 71 858–865. 10.1093/gerona/glv307 26809496 PMC4906327

[B30] Zhang C. Zhu J. Zhou Y. Thampatty B. P. Wang J. H. (2019). Tendon stem/progenitor cells and their interactions with extracellular matrix and mechanical loading. *Stem Cells Int.* 2019:3674647. 10.1155/2019/3674647 31737075 PMC6815631

[B31] Zhang J. Li F. Williamson K. M. Tan S. Scott D. Onishi K. (2021). Characterization of the structure, vascularity, and stem/progenitor cell populations in porcine Achilles tendon (PAT). *Cell Tissue Res.* 384 367–387. 10.1007/s00441-020-03379-3 33496880 PMC8154658

[B32] Zhang J. Pan T. Liu Y. Wang J. H. (2010). Mouse treadmill running enhances tendons by expanding the pool of tendon stem cells (TSCs) and TSC-related cellular production of collagen. *J. Orthop. Res.* 28 1178–1183. 10.1002/jor.21123 20225313

[B33] Zhang J. Wang J. H. (2010a). Characterization of differential properties of rabbit tendon stem cells and tenocytes. *BMC Musculoskelet. Disord.* 11:10. 10.1186/1471-2474-11-10 20082706 PMC2822826

[B34] Zhang J. Wang J. H. (2010b). Mechanobiological response of tendon stem cells: implications of tendon homeostasis and pathogenesis of tendinopathy. *J. Orthop. Res.* 28 639–643. 10.1002/jor.21046 19918904

[B35] Zhang J. Wang J. H. (2010c). Production of PGE(2) increases in tendons subjected to repetitive mechanical loading and induces differentiation of tendon stem cells into non-tenocytes. *J. Orthop. Res.* 28 198–203. 10.1002/jor.20962 19688869

[B36] Zhang J. Wang J. H. (2013). The effects of mechanical loading on tendons–an in vivo and in vitro model study. *PLoS One* 8:e71740. 10.1371/journal.pone.0071740 23977130 PMC3747237

