# Supplementary figures and images for: Mechanical Overloading Induced-Activation of mTOR Signaling in Tendon Stem/Progenitor Cells Contributes to Tendinopathy Development
Source: Front Cell Dev Biol. 2021 Jul 12;9:687856. doi: 10.3389/fcell.2021.687856 (PMC8311934; doi:10.3389/fcell.2021.687856)

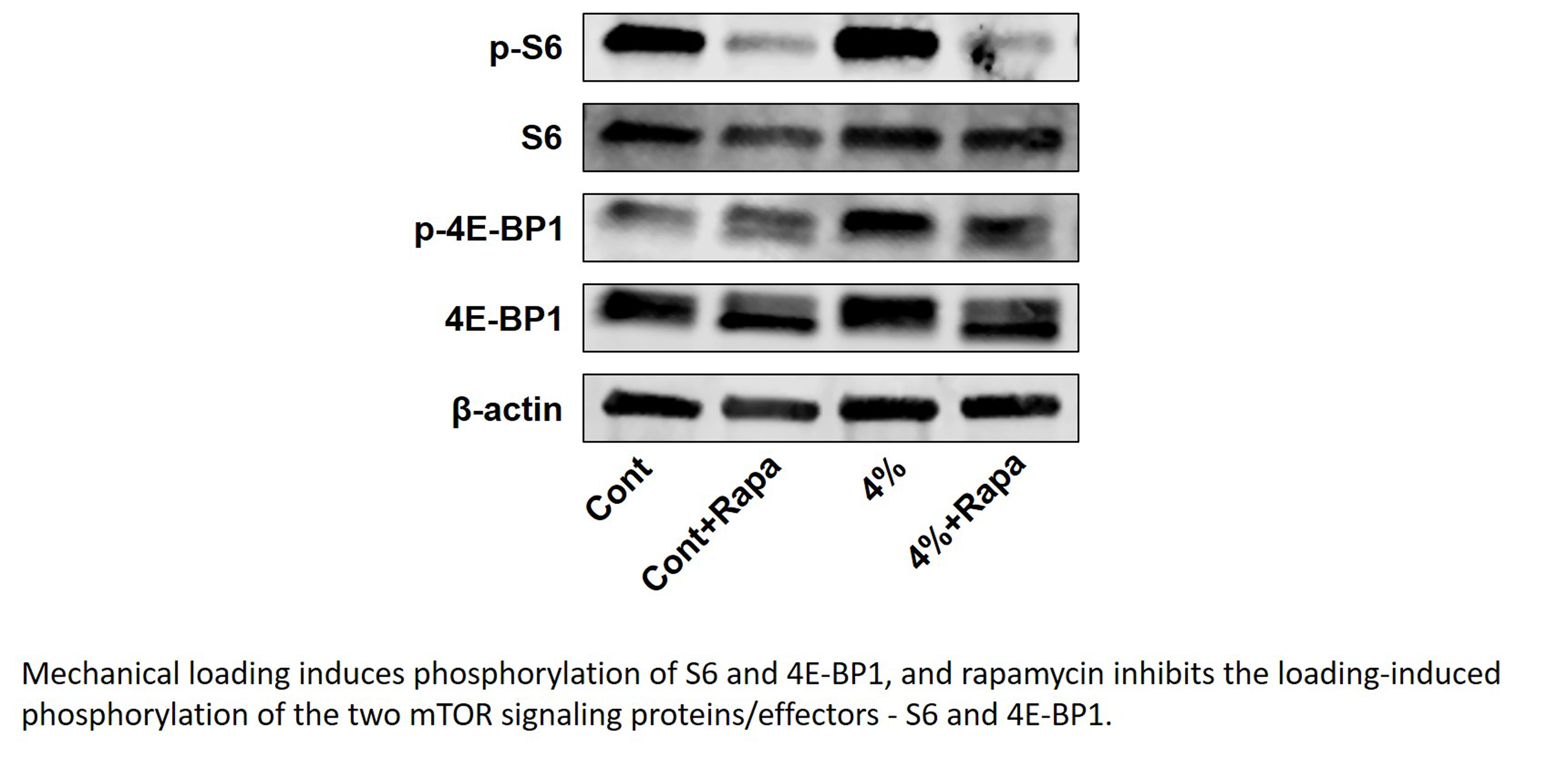

Supplement: Supplementary Figure 1 — Rapamycin inhibits the phosphorylation of both S6 and 4E-BP1. [file Image_1.TIF]
